# Approaches and impact of non-academic research capacity strengthening training models in sub-Saharan Africa: a systematic review

**DOI:** 10.1186/s12961-015-0017-8

**Published:** 2015-06-09

**Authors:** Lambert Mugabo, Dominique Rouleau, Jackline Odhiambo, Marie Paul Nisingizwe, Cheryl Amoroso, Peter Barebwanuwe, Christine Warugaba, Lameck Habumugisha, Bethany L. Hedt-Gauthier

**Affiliations:** Partners In Health-Inshuti Mu Buzima, P.O. Box 3432, Kigali, Rwanda; Global Health Corps, One Penn Plaza, Suite 6271, New York, NY 10119 USA; Department of Global Health and Social Medicine, Harvard Medical School, 641 Huntington Avenue, Boston, MA 02115 USA

**Keywords:** Capacity strengthening, Capacity building, Outcomes, sub-Saharan Africa, Systematic review

## Abstract

**Background:**

Research is essential to identify and prioritize health needs and to develop appropriate strategies to improve health outcomes. In the last decade, non-academic research capacity strengthening trainings in sub-Saharan Africa, coupled with developing research infrastructure and the provision of individual mentorship support, has been used to build health worker skills. The objectives of this review are to describe different training approaches to research capacity strengthening in sub-Saharan Africa outside academic programs, assess methods used to evaluate research capacity strengthening activities, and learn about the challenges facing research capacity strengthening and the strategies/innovations required to overcome them.

**Methodology:**

The PubMed database was searched using nine search terms and articles were included if 1) they explicitly described research capacity strengthening training activities, including information on program duration, target audience, immediate program outputs and outcomes; 2) all or part of the training program took place in sub-Saharan African countries; 3) the training activities were not a formal academic program; 4) papers were published between 2000 and 2013; and 5) both abstract and full paper were available in English.

**Results:**

The search resulted in 495 articles, of which 450 were retained; 14 papers met all inclusion criteria and were included and analysed. In total, 4136 people were trained, of which 2939 were from Africa. Of the 14 included papers, six fell in the category of short-term evaluation period and eight in the long-term evaluation period. Conduct of evaluations and use of evaluation frameworks varied between short and long term models and some trainings were not evaluated. Evaluation methods included tests, surveys, interviews, and systems approach matrix.

**Conclusions:**

Research capacity strengthening activities in sub-Saharan Africa outside of academic settings provide important contributions to developing in-country capacity to participate in and lead research. Institutional support, increased funds, and dedicated time for research activities are critical factors that lead to the development of successful programs. Further, knowledge sharing through scientific articles with sufficient detail is needed to enable replication of successful models in other settings.

## Background

High quality research is essential to identify and prioritize health needs and to develop appropriate strategies to improve health outcomes [1]. However, despite the increase of publications from Africa during the past two decades [2], the representation of Africa in global research output is disproportionately low. For example, between 1997 and 2006, only 7 % of global tuberculosis research output came from Africa despite the region having the highest tuberculosis case rates in the world [3]. In 2004, research about Africa represented less than 1 % of scientific publications [4], growing gradually to 10 % as of 2011 [5].

In the last decade, the international call for developing research capacity in sub-Saharan Africa has grown [4, 5]. Opportunities to support individuals pursuing academic studies and fellowships at academic institutions have increased [6, 7]. However, there are several limitations to academic programs as the sole means for capacity strengthening in sub-Saharan Africa – they can be long to complete and expensive and present a potential risk of drawing national researchers from program settings into academia, especially if no strong partnerships exist between academia and local programs [3]. Further, academic research tends to miss operational perspectives from programs [3]. To overcome these limitations and as a complement to these academic programs, local organizations/institutions across Africa, often in partnership with institutions from developed countries, have implemented short trainings targeting specific research competencies of health program staff. The term ‘non-academic’ is used throughout this paper to refer to training programs that do not lead to formal academic qualifications, although they may use academic training staff and/or infrastructure.

Strengthening research capacity in non-academic settings encompasses a variety of activities, including trainings to support individuals to acquire research skills in addition to developing research infrastructure at an institutional level, creating research partnerships/networks, and providing individual support and mentorship [8]. In this paper, we focus specifically on the training activities in the research capacity strengthening programs. The goals, approaches, target audience, and effectiveness of the skill-specific research trainings in sub-Saharan Africa vary widely. However, there are few peer-reviewed published descriptions of these activities to support the replication or adaptation of such programs in other locations. The objectives of this systematic review are therefore 1) to describe the different approaches to research capacity strengthening in sub-Saharan Africa beyond academic programs, 2) to assess methods used to evaluate research capacity strengthening activities and summarize their results, and 3) to learn about challenges to research capacity strengthening and strategies/innovations to overcome those challenges. This review will contribute to research capacity strengthening efforts by providing insights from different approaches that could be applied to other locations and to encourage more complete reporting of such initiatives.

## Methods

### Identification of data sources

**Fig. 1:**
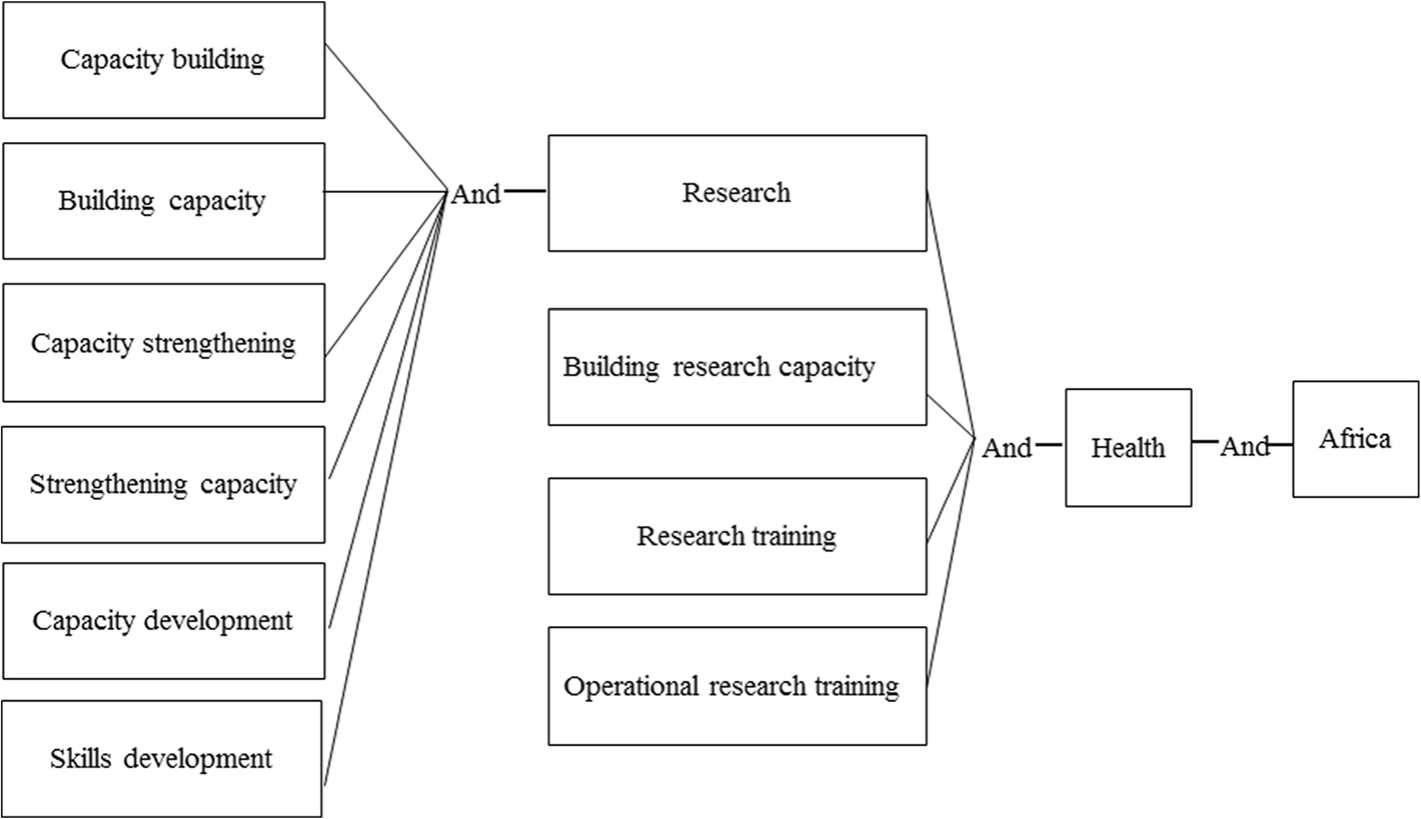
Search terms for systematic review

The PubMed database was searched by the principal investigator (LM) for articles describing research capacity strengthening training activities in Africa. The following search terms were used (illustrated in Fig. 1): words that indicate an increase in competency (“building”, “development”, “strengthening”, and “training”) combined with “capacity” as well as the terms “Africa” and “health” and “research”. Further search criteria were 1) papers published between 2000 and 2013 and 2) both abstract and full paper available in English. The results were saved into a Mendeley library.

### Study selection

**Fig. 2:**
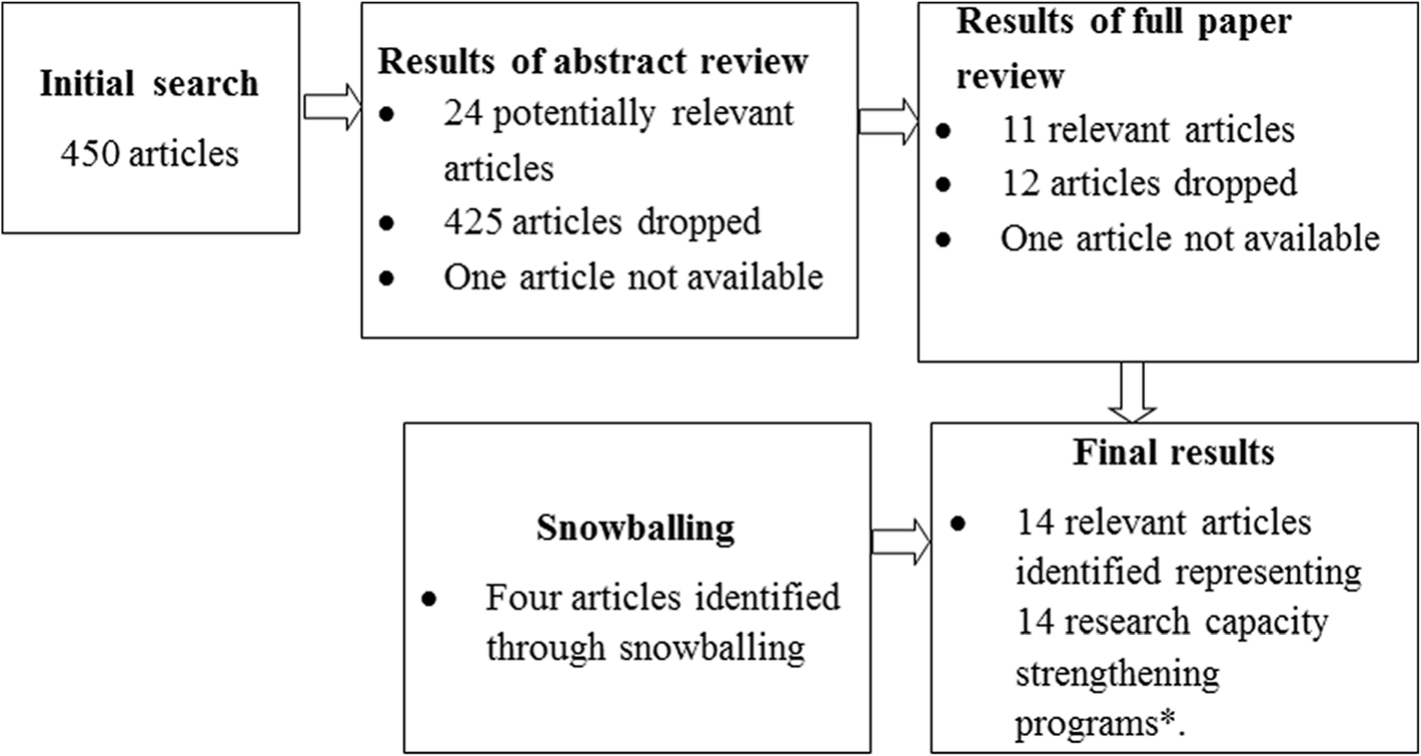
Search and selection process in the review on research capacity strengthening in sub-Saharan Africa

The titles and abstracts were reviewed by the principal investigator (LM) to ensure they met the following inclusion criteria: 1) research capacity strengthening training activities are explicitly described, including information on program duration, target audience, and immediate program outputs and outcomes, 2) all or part of the training program took place in sub-Saharan African countries, and 3) the training activities are not a formal academic program. When all criteria were met, or more information was needed, articles were retained for full text review (Fig. 2). Articles were also assessed after full text review and dropped if not meeting all eligibility criteria. Articles not captured in the original search were added, either because they were known to the authors or were identified through a snowballing process of reviewing the reference list of retained articles.

### Data extraction and analysis

Two independent reviewers extracted data from the full text articles, which was captured in a three-part data collection form. The form was developed based on the research team’s experience in conducting research strengthening activities and adapted based on themes that emerged during the review of articles. The first part covered program description information, including name of the program, program duration, target audience, objectives of the training, frequency of the training, qualification of the trainers, resources required for the training, and where the training took place. The second part used Cooke’s evaluation framework to assess the effectiveness of the trainings [9]. Cooke’s framework was chosen because it comprehensively describes the indicators for individual training in research capacity building based on six principles: research skills, practice implications, partnerships, dissemination, infrastructure, and sustainability. We grouped the trainings based on their evaluation period. The short term evaluation period was defined as evaluations conducted up to 18 months after the training. The long-term evaluation period was any period greater than 18 months.

Finally, data were extracted on challenges faced, innovations used, and recommendations proposed for future programs. Data extractions from both reviewers were entered into a Microsoft Access database and compared for consistency. When inter-reviewer discrepancies were found, they were resolved by a third party review of the paper.

## Results

The search resulted in 495 articles, of which 450 were retained following the removal of duplicates (Fig. 2). Based on abstract review, 24 articles were classified as potentially relevant, 425 were dropped, and one abstract was not available. Full text review of the 24 articles yielded 11 relevant texts. The same training program was presented in two of these articles and so only the more recent and relevant publication was retained. Four additional articles were identified through the snowball process, resulting in 14 relevant articles in total.

### Description of the research capacity strengthening programs

The 14 research capacity strengthening trainings described in these papers include four which started in the 1990s [10–13] and the rest from 2000 or later [14–21] (Table 1). Most programs took place in the southern African region [10, 12, 13, 20], followed by Uganda [16, 18]. Malawi [21], Nigeria [14], Cameroon [17], and the Democratic Republic of Congo [19] were also represented. For two trainings, some of the training activities took place in the United States of America [15] or Europe [11]. Most of the training activities were implemented multiple times [10–16, 20, 22].

**Table 1:** Characteristics of approaches in the review of research capacity strengthening activities in sub-Saharan Africa

Reference	Location of the training	Training goal and specific competencies	Target trainees	Faculty/trainers	Structure, duration of the training activities, and frequency of offering	Funding and partnership	Technical support/follow-up during the program
Adams et al. 2003 [10]	South Africa	Goal: Provide skills for health service evaluation Competencies: Research ethics, research methods, data capture and analysis, research protocol and report writing	300 trainees, all African: Health service middle managers and MSc students within the country	In-country based faculty	Training activities lasted 2 weeks and offered 13 times during 1992–2001	Total funds: Not reported (materials expenses mentioned) Funding source and partnerships: Not reported	No
Ajuwon and Kass 2008 [14]	Nigeria	Goal: To develop the capacity of academic staff to conduct ethically acceptable research involving human population Competencies: Research ethics	133 trainees, all African: Clinical staff from College of Medicine and researchers from NGOs and IRB	Locally based seven resource persons with experience	Training activities lasted 21 hours spread over 3 days and offered three times during 2003–2004	Total funds: Not reported (materials expenses mentioned) Funding source and partnerships: NIH, Wellcome Trust, Fogarty International Center	No
Ali et al. 2012 [15]	Blended between USA and the country of origin	Goal: Training on research ethics to health professionals. Competencies: Ethics and research methods	28 trainees, all African: Researchers mainly from Eastern Africa, most of which had graduate degrees with research experience, health professionals, ethics committee members, journalists and scientists	Associate faculty from JHU, the NIH, associated research ethics programs, and African professionals	Program lasted 1 year, 6 months of courses and seminars, IRB involvement and development of field project and 6 months for practicum and was offered multiple times during 2001–2009	Total funds: Not reported (materials, flight expenses mentioned) Funding source and partnerships: NIH	Continuous mentorship from JHU and African faculty, biannual reunion meeting of alumni and faculty for networking and exchanging ideas
Matovu et al. 2013 [16]	Uganda	Goal: Strengthen the capacity of M&E and continuous quality improvement using work-based training model Competencies: Data collection, data analysis, project proposal, report writing and M&E	143 trainees, all African: Mid- and senior-level managers, coordinators and supervisors within the country	MakSPH faculty and external facilitators	Training activities lasted 5 weeks of face-to-face sessions and 6 months of field activities between 2nd and 3rd modules and was offered multiple times during 2008–2011	Total funds: $2500 for project implementation Funding source and partnerships: CDC	Ongoing technical support from an academic mentor over the program
Mbuagbaw et al. 2011 [17]	Cameroon	Goal: Training on how to initiate and complete systematic reviews Competencies: Design, analysis and interpretation of systematic review and meta-analysis	15 trainees, all African: University lecturers and researchers within the country	Cochrane Review authors and researchers from Africa and Chile	Training activities lasted 4 days of face-to-face sessions and was offered once in 2011	Total funds: Not reported	No
						Funding source and partnerships: Cochrane Collaboration, South African Medical Research Council, Yaunde Central Hospital, and Global Health Research Initiative	
Njie-Carr et al. 2012 [18]	Uganda	Goal: Research capacity building to assess implementation of mobile service for HIV intervention Competencies: Research ethics, research methods, data collection	14 trainees, all African: Employees and volunteers at Reach Out, a large HIV/AIDS care and service program in Kampala	Five authors in total from fields of medicine, nursing, psychology, biology, and public and international health	Training activities lasted 6 days of intensive didactic training and 4 weeks of field activities and offered once in 2010	Total funds: Not reported (software fees mentioned) Funding source and partnerships: RO, Makerere University, and Johns Hopkins	Continuous mentorship from trainers over the program
Tshikala et al. 2012 [19]	Democratic Republic of Congo	Goal: Train on research bioethics through ancillary care Competencies: Research ethics	30 trainees, all African: Members of CIBAF, faculty from universities, members of clinical ethics committee, representatives of NGOs, paediatric clinics and National AIDS Control Program, members of UNC/DRC	Members of GIRIE, CIBAF and KSPH faculty	Training activities lasted 3 days of formal presentations and discussion and offered once	Total funds: Not reported (materials expenses mentioned) Funding source and partnerships: NIH/Fogarty International Center	No
Williams et al. 2010 [20]	South Africa	Goal: Increase research training and utilization of existing datasets Competencies: Data management and analysis	55 trainees, 40 African: Masters and PhD students from Wits, CU, Brown University and researchers from APHRC	CU researchers, Institutional faculty from Wits, CU, Brown University and APHRC	Training activities lasted 3 weeks of lectures, guided exercises, and research projects and was offered three times during 2006–2008	Total funds: Not reported Funding source and partnerships: Wit School of Public Health, University of Colorado, African Population Studies Research and Training Program	No
Buist and Parry 2013 [11]	Multiple countries in sub-Saharan Africa	Goal: increasing local and national research capacity Competencies: Research ethics, research methods, data collection and analysis, research protocol and writing report	1015 trainees, 112 African: Practicing and academic physicians and public health professionals across Africa	Local and international behavioural, epidemiologic, public health, and statistical researchers	Five courses structured as a ladder with each one lasting 5 to 5 1/2 days	Total funds: Not reported (material expenses mentioned) Funding source and partnerships: CDC, USAID, ATS	Students receive mentoring following the course, incentives to support research projects and editorial assistance are provided
					Courses involved lectures, small groups to develop protocol, and daily homework and was offered multiple times during 1994–2013		
Chilengi et al. 2013 [22]	Web-based targeting African researchers	Goal: Complement other forms of learning though online training on health research ethics and good clinical practice Competencies: Health research ethics and good clinical practice	1155 trainees, 958 African: Researchers from multiple countries	Trainers or practitioners of research ethics within Africa	Training lasted 100 days	Total funds: Not reported (software expenses mentioned) Funding source and partnerships: EDCPT and AMANET	No
Harries et al. 2003 [21]	Malawi	Goal: Operational research training for TB related research Competencies: Data collection and data analysis, research protocol and manuscript writing	25 trainees, all African: TB officers from district and mission hospitals within the country	NTP facilitators from the Central Unit and Regional TB offices	Training activities lasted 1 1/2 days of seminar on OR and development of protocol, 6 months of field work, 1 day workshop of data analysis and writing a paper and was offered once in 2000	Total funds: Not reported (financial incentives mentioned) Funding source and partnerships: National governments and District TB units	Field supervisory visits are carried out once or twice yearly by central unit to assess data collection
Laserson et al. 2005 [12]	South Africa then expanded to regional course	Goal: building capacity in basic epidemiology and operations research Competencies: Qualitative methods, study design, data collection and data analysis, research protocol and manuscript writing	149 of various nationalities: National, provincial, and district-level NTP managers and TB laboratory directors and staff	International and in-country epidemiologists and TB experts	Training activities lasted 6 days, involving lectures, field exercises, development of OR protocol and 12 months of field implementation of the protocol, often in groups and was offered nine times during 1997–2004	Total funds: US$2000 – 20,000 Funding source and partnerships: NTP in various countries, USAID, WHO, CDC, Pan American Health Organization	Technical assistance is provided during field activities including further training
Varkevisser et al. 2001 [13]	Southern African Region	Goal: increase national capacity for operational research Competencies: Data collection and data analysis, research protocol and manuscript writing	1159 trainees, all African: Higher and middle level health workers from provincial and district level	University staff, senior health trainers and higher-level health staff who have completed an HSR methodology before	Training activities lasted 14–16 days of workshop to develop research proposal, 6 months to collect data, followed by 12–14 days of data analysis and writing a report and was offered 50 times during 1987–1997	Total funds: USD$5000–$8000 per study	Institutional support from local authority is sought through special meetings at national and inter-country level organized at regular intervals (2–3 years)
						Funding source and partnerships: WHO, The Netherlands Ministry of Development and Cooperation, USAID, IDRC, Norad	
Zachariah et al. 2011 [23]	Multiple countries	Goal: building leadership in operational research Competencies: Research questions and protocol development, data management and analysis, paper writing	Number of trainees not reported: Persons who work within disease programmes and who are committed and have opportunities to carry out operational research	International	Training activities lasted 3 weeks spread over 9 months with significant intervals between modules and frequency of offering is not reported	Total funds: $500–$1500 small grants Funding source and partnerships: The Union/MSF	Technical support throughout the program

*AMANET* African Malaria Network Trust, *APHRC* African Population and Health Research Center, *ATS* American Thoracic Society, *CDC* US Centers for Disease Control, *CIBAF* Centre Interdisciplinaire de Bioethique pour L’Afrique Francophone, *CU* University of Colorado-Boulder, *DRC* Democratic Republic of Congo, *EDCPT* European Developing Countries Clinical Trials Partnership, *GIRIE* Groupe Interproject de Reflexion et d’Intervention en Ethique, *IDRC* International Development Research Centre, *IRB* Institutional Review Board, *JHU* Johns Hopkins University, *KSPH* Kinshasa School of Public Health, *M&E* Monitoring and Evaluation, *MakSPH* Makerere University School of Public Health, *MSF* Médecins Sans Frontières, *NGO* Non-Governmental Organization, *NIH* National Institutes of Health, *Norad* Norwegian Agency for Development Cooperation, *NTP* National TB Control Program, *OR* Reach Out, *TB* Tuberculosis, *UNC* University of North Carolina, *USAID* United States Agency for International Development, *WHO* World Health Organization

### Research competencies covered

Of the 14 training programs identified, eight covered multiple competencies [10–13, 16, 18, 21, 23], while six focused on a single research competency [14, 15, 17, 19, 20, 22]. The most covered competencies included research ethics [10, 11, 14, 15, 18, 19, 22], research methods [10–12, 15, 18], data collection [10–13, 16, 18, 21], data analysis [10–13, 16, 17, 20, 21, 23], research protocol/research design [10–13, 16, 21, 23], and writing a report or manuscript [10–13, 16, 21, 23]. Of six training programs focusing on a single research competency, four focused on research ethics [14, 15, 19, 22] and one focused on systematic reviews [17], whereas the other one targeted data management and analysis skills [20].

### Target audience and trainers

In total, 4136 people were trained of which at least 2939 were from Africa. Trainees in these programs were of very different backgrounds and qualifications. Participants included clinical staff, health officers and managers working within health programs, university students and faculty, and experienced researchers. Generally, participants were selected based on their potential to influence health systems and management processes, ability to conduct research activities, and their involvement or expertise in the field. Only two training programs had a rigorous selection process whereby criteria, such as years of experience in research ethics, number of publications, institutional support, and personal commitment, were considered [15, 23]. For 11 of the 14 programs, participants were from the country where the training took place. However, three of the programs required out-of-country travel of the participants to the training site. For these, one was in sub-Saharan Africa [10] and two in Europe or USA [15, 23].

Specific details about the qualifications of trainers were not reported. Most of them were individuals with experience and expertise in the area of interest, either based in the country or brought in through a partnership as an international expert. They included faculty from universities, researchers, and practitioners in a given field.

### Structure, duration of the trainings and follow-up

Structure and duration of research strengthening activities outside academic settings vary widely. Five of 14 training programs [10, 14, 17, 19, 20] only featured face-to-face sessions conducted over a short period of time; mostly less than a week. Seven training programs featured both face-to-face sessions and practicums. Of these, four had face-to-face sessions spread over a longer period with intervals of field activities taking place in between [13, 16, 23]. Face-to-face sessions took at least 3 weeks, whilst one training program featured short classes of 2 1/2 days [21]. Three training programs mixing face-to-face sessions with practicums conducted the practicum after the face-to-face sessions. Two had 6 days of classes [12, 18], whilst another one had a longer period of face-to-face classes [15] and practicum ranges between 1 [18] and 12 months [15]. Further, one training program had five courses conducted over 5 days each, structured as a ladder [11], where success at a lower level determined who moved up to the next. Finally, one training program was web-based [22], taking 100 days to complete. Seven training programs provided follow-up to their trainees [11, 12, 15, 16, 18, 21, 23] in terms of ongoing mentorship, onsite technical assistance, and supervisory visits.

### Evaluation

Of the 14 training programs, six fell in the category of short-term evaluation period [14, 17–19, 21, 22] and eight in the long-term evaluation period [10–13, 15, 16, 20, 23]. For the training programs with a short-term evaluation period, one was not evaluated [19] and only one (16.7 %) used a recognized framework for evaluation [18]. These training programs used quantitative evaluation methods, mainly surveys and tests. Of the training programs with long-term evaluation periods, 37.5 % (n = 3) used a framework [10, 15, 16]. All of these training programs were evaluated, using quantitative or qualitative methods including interviews, surveys, and systems approach framework.

#### Training programs with short term evaluation period

All training programs reported an increase in research knowledge and skills (100 %; Table 2). More than half of the training programs (50–67.7 %), reported the involvement of practitioner and program staff in the training, the relevance or use of training related research in practice, and the existence of inter-professional linkages. None of these training programs, however, reported on or used impactful dissemination (publication, conferences, workshop presentations, changes in policy and practice) as a key principle in research capacity strengthening. Further, there was no information about conduct of research after training, patient centred outcome measures, access to funding post training, availability of protected research time, or existence of mentorship and supervision structures.

**Table 2:** Evaluation details of six trainings with short evaluation periods

		Short term evaluation period trainings						
Indicators		Tshikala et al. 2012 [19]	Chilengi et al. 2013 [22]	Mbuagbaw et al. 2011 [17]	Ajuwon and Kass 2008 [14]	Njie-Carr et al. 2012 [18]	Harries et al. [21] 2003	Percentage of studies reporting on indicators
Study’s evaluation approach								
Evaluation framework used		Not evaluated	NR	NR	NR		NR	16.7
Evaluation method (qualitative/quantitative)			Quant	Quant	Quant	Quant	Quant	
Details (satisfaction survey/self-reported changes/pre-post skills test/research outputs survey)			Test Scores	Satisfaction surveys	Pre/Post Tests	Surveys	Program Data	
Program evaluation period months			NR	0.13	1	1	15	
Reviews measures of effectiveness of RCS, based on Cooke’s framework								
Improved confidence and skills	Evidence of knowledge and skills developed (e.g., improved post-test scores)	Yes	Yes	Yes	Yes	Yes	Yes	100.0
	Evidence of confidence building (e.g., trainees become trainers; obtained research-related jobs)	NR	NR	NR	NR	Yes	NR	16.7
	Research undertaken after training (e.g., involvement in subsequent research)	NR	NR	NR	NR	NR	NR	0.0
Research is close to practice	Practitioner and program staff involvement (e.g., nurse, manager trainees)	Yes	No	No	No	Yes	Yes	50.0
	Research relevant to or used in practice (e.g., reported changes in practice)	Yes	NR	NR	NR	Yes	Yes	50.0
	Patient centred outcome measures used	NR	NR	NR	NR	NR	NR	0.0
	Action oriented methodologies used (e.g., research done on quality care)	NR	NR	NR	NR	NR	Yes	16.7
Research enhanced by partnerships	Between novice and experienced researchers	No	NR	No	No	No	No	0.0
	Inter-professionals linkages (e.g., between researchers, policy makers, different disciplines)	Yes	NR	No	Yes	Yes	Yes	66.7
Impactful dissemination	Publications in peer-reviewed journals	No	NR	NR	NR	NR	No	0.0
	Conference/workshop presentation	No	NR	NR	NR	NR	NR	0.0
	Evidenced of applied research findings (e.g., changes in policy/practice reported)	No	NR	NR	NR	NR	NR	0.0
Continuity and sustainability	Successful access of funding (grants/fellowships)	No	NR	NR	NR	NR	NR	0.0
	Enduring collaborations (e.g., relationship building between involved institutions to promote individual training)	Yes	NR	NR	NR	NR	NR	16.7
	Continued mentorship and supervision	No	NR	NR	NR	Yes	Yes	33.3
Infrastructure for research	Institutional support for undertaking research	Yes	NR	NR	NR	NR	Yes	33.3
	Protected research time	No	NR	NR	NR	NR	NR	0.0
	Budget line	NR	NR	NR	NR	NR	Yes	16.7
	Mentorship and supervision structures	No	NR	NR	NR	NR	NR	0.0

*NR* Not reported

#### Training program with long-term evaluation period

All training programs reported on an increase in research knowledge and skills and research undertaken after training (100 %; Table 3). More than half of these trainings (50–87.5 %), reported evidence of confidence building among trainees, the involvement of practitioner and program staff in the training, the relevance or use of training-related research in practice, the existence of inter-professional linkages, publications in peer-reviewed journals, evidence of applied research findings, continued mentorship and supervision, and enduring collaborations. None of these training programs either reported on or used availability of protected research time, budget lines, or existence of mentorship and supervision structures.

**Table 3:** Evaluation details of eight trainings with long evaluation periods

		Long term evaluation period								
Indicators		Matovu et al 2013 [16]	Williams et al. 2010 [20]	Adams et al 2003 [10]	Laserson et al. 2005 [12]	Ali et al. 2012 [15]	Varkevisser et al. 2001 [13]	Buist and Parry 2013 [11]	Zachariah et al. 2011 [23]	Percentage of studies reporting on indicators
Study’s evaluation approach										
Evaluation framework used		Yes	NR	Yes	NR	Yes	NR	NR	NR	37.5
Evaluation method (qualitative/quantitative)		Qual	Mixed	Qual	Quant	Quant	Quant	Quant	NA	
Details (Satisfaction survey/self-reported changes/pre- and post-skills test/research outputs survey)		Interviews	Program data	Focus Group	Questionnaire	Database and reports	Meetings and reports	Email Survey	NA	
Program evaluation period months		36	48	60	84	96	168	204	NR	
Reviews measures of effectiveness of RCS, based on Cooke’s framework										
Improved confidence and skills	Evidence of knowledge and skills developed (e.g., improved post-test scores)	Yes	Yes	Yes	Yes	Yes	Yes	Yes	Yes	100.0
	Evidence of confidence building (e.g., trainees becomes trainers; obtained research-related jobs)	Yes	Yes	NR	NR	Yes	Yes	Yes	NR	62.5
	Research undertaken after training (e.g., involvement in subsequent research)	Yes	Yes	Yes	Yes	Yes	Yes	Yes	Yes	100.0
Research is close to practice	Practitioner and program staff involvement (e.g., nurse, manager trainees)	Yes	No	Yes	Yes	Yes	Yes	Yes	Yes	87.5
	Research relevant to or used in practice (e.g., reported changes in practice)	Yes	NR	Yes	Yes	NR	Yes	Yes	Yes	75.0
	Patient-centred outcome measures used	Yes	No	NR	NR	NR	NR	No	NR	12.5
	Action oriented methodologies used (e.g., research done on quality care)	Yes	NR	NR	NR	NR	NR	Yes	NR	25.0
Research enhance by partnerships	Between novice and experienced researchers	Yes	Yes	No	NR	No	NR	No	No	25.0
	Inter-professional linkages (e.g., between researchers, policy makers, different disciplines)	Yes	Yes	No	NR	Yes	No	Yes	No	50.0
Impactful dissemination	Publications in peer-reviewed journals	NR	NR	NR	NR	Yes	Yes	Yes	Yes	50.0
	Conference/workshop presentation	Yes	NR	NR	NR	Yes	NR	Yes	NR	37.5
	Evidenced of applied research findings (e.g., changes in policy/practice reported)	Yes	NR	NR	Yes	NR	Yes	Yes	Yes	62.5
Continuity and sustainability	Successful access of funding (grants/fellowships)	NR	NR	NR	NR	Yes	NR	Yes	Yes	37.5
	Enduring collaborations (e.g., relationship building between involved institutions to promote individual training)	Yes	Yes	NR	NR	Yes	Yes	Yes	NR	62.5
	Continued mentorship and supervision	Yes	NR	No	Yes	Yes	NR	Yes	Yes	62.5
Infrastructure for research	Institutional support for undertaking research	Yes	NR	NR	NR	NR	Yes	NR	NR	25.0
	Protected research time	NR	NR	NR	NR	NR	NR	NR	NR	0.0
	Budget line	NR	NR	NR	NR	NR	NR	NR	NR	0.0
	Mentorship and supervision structures	NR	NR	NR	NR	NR	NR	NR	NR	0.0

### Challenges, innovations and recommendations

This review identified major themes regarding challenges to research capacity strengthening activities and suggested corresponding innovations and recommendations to address the challenges (Table 4). Common challenges to capacity strengthening were lack of mentorship and institutional support [10, 13, 16, 18, 20, 21, 23]; insufficient time for research activities and drop out [10, 16, 18, 20, 21]; lack of sufficient budget for research activities [11, 13, 18, 23]; poor research infrastructure [12, 13, 17, 18, 23]; and difficulty in publishing in international journals [11, 21, 23]; three papers did not report any challenges [16, 18, 21].

**Table 4:** Challenges, recommendations and innovations regarding research capacity strengthening activities

Challenge	As faced by trainees	As faced by organizers/facilitators	Innovations/recommendations
Lack of mentorship and institutional support	Participants’ initiatives blocked by managers [10]	Lack of strategies encouraging recent trainees to apply new learning within the services [10] Difficulty getting buy-in from institutions [16, 23]	Provide mentorship to participants by managers to enhance application of acquired skills on the job [10]
	Drop out from training program because of no mentorship [16, 18, 20]		
			During application approvals, organizational commitment to in-service training for capacity development [10]
	Delay in completing research projects because of no mentorship [16]	Weak co-ordination due to incompetency of leaders [13]	Support professional network and alternative communication pathways to
			improve intra- and inter-program collaboration [15]
	Lack of communication between participants and supervisors [21]		
			Engage with institutions from the beginning and get commitment from program leadership [16, 18]
			Sensitize policy-makers and health managers through special meetings [13]
Poor research infrastructure	Poor internet [17]	Poor internet [17]	Improve internet access [17, 23]
	Inadequate space and lack of equipment [18, 23]	Difficulty in securing adequate space for research activities [12, 13, 18, 23]	Provide budget lines dedicated for improving research infrastructure [23]
Insufficient time for research and program dropouts	Trainees get absorbed into routine work and responsibilities [16]	Loss of trainees through dropout [16, 18, 20]	Conducting training activities at the workplace
	Trainees take jobs with other institutions [16]	Trainers do not have resources nor authority to conduct effective follow-up within workplace [10] Mismatches between participants’ capabilities and training priorities [21]	Increase time allocated to research activities [18, 23]
			Suitable training schedule [18]
			Establish strong selection criteria to minimize dropouts [23]
			Add distance learning to face-to-face classes
			Provide support supervision to trainees by program staff and/or mentors [16]
Lack of funds for research activities	Lack of resources to conduct research activities [11, 13, 23]	Dependence on external institutions or donors for funding [13]	Build more resources for funding [11]
			Embed research agenda into health program [21]
	Difficulty in accessing training location [18]		Develop strong institutional infrastructure (administrative leadership) [18]
			Integrate courses into existing curriculum [16, 20]
Difficulty in publishing papers in international journals	Difficulties in publishing in international journals [11, 21, 23]		Mentor on publication process [11]
			Strengthen selection criteria to get strong candidates
			Explore other opportunities such as publishing in local journals and presenting at local meetings [21]
			Provide further training [21]
Language barriers and differences in educational levels	Trainees face communication challenges [18]	Difficult to manage a group of different levels of education [21] and/or speaking different languages [18]	Strategic groupings of participants with similar skill levels [21]

Challenges faced by participants are distinguished to those faced by facilitators and organizers. On the one hand, participants who lack support and mentorship from supervisors and managers are more likely to drop out of the training or their research projects are likely to be delayed. On the other hand training organizers and facilitators find it difficult when participants are pulled out of the training because of other work responsibilities, particularly when training organizers and the organization where a participant works do not have a memorandum of understanding. Infrastructural challenges such as poor internet and inadequate space and equipment affect both participants and facilitators’ performances. Further, when participants have heavy workloads they are likely to drop out of the training, thus affecting trainers and organizers. A lack of funding implies that any research requiring funds will not be performed and training activities could be hampered, for example, when participants need transport and do not have money. For organizers, a lack of funding could mark the end of training activities since they face shortages of materials, facilitators, and poor infrastructure.

Various recommendations and innovations are proposed to address the challenges to research capacity strengthening. Institutional support and mentorship is achieved in different ways such as provision of mentorship and supervision visits by programme managers [10, 16], developing strong professional network [15], and seeking commitment from stakeholders [13, 16, 18]. Increased time for research [18, 23], suitable training schedule [18], and creating web-based training helps to tackle the challenge of insufficient time. Building more funding resources for research activities [11], embedding research into a health program [21], and integrating courses into existing curriculum [16, 20] are recommended as strategies to address the lack of funding. The challenge of publication could be addressed through provision of mentorship on publication process [11] and finding other means of dissemination than international journals [21], for example, through special meetings with stakeholders. Provision of further training to improve writing skills of young researchers would increase the likelihood of having a manuscript accepted for publication.

## Discussion

In this systematic review, we identified 14 papers that describe research capacity strengthening activities outside of formal academic programs in sub-Saharan Africa. We found that training programs generally fell into two categories: longer training programs covering multiple competencies and shorter training programs targeting a single research competency. Generally, shorter programs did not have practicum projects as part of the training nor did they provide mentorship/support post-training. These two features make such programs less expensive and less time consuming and therefore more feasible for many settings. However, though their contribution to the increase in research skills and knowledge is recognized, we found little evidence that links these programs to the research projects conducted. Further, offering trainings that focus on narrow competencies would then require multiple trainings to enable participants to take a research question through to publication, if this is the intended goal.

Alternatively, training programs which are more comprehensive yield better outcomes in terms of the number of research projects conducted and resulting publications. They are offered over a longer period and often require ongoing mentorship/support. The demands on both human and financial resources make such trainings more expensive and time consuming and therefore less accessible to many organizations.

Most of the studies in our review did not report on the program implementation costs. When reported, these costs varied widely, between $500 and $20,000 per project, depending on scope of the project, location, and duration of training. While actual costing of programs is difficult, reporting of the estimated expenditure are important to other people planning these training activities, particularly because resource allocation is among the major barriers in research capacity strengthening activities. Except for one program that was a national program [21], programs primarily relied on North–South partnerships for funding, highlighting the need for strengthening partnerships with more focus on South research agenda [24], as well as galvanizing national resources and increasing South–South research collaboration.

In addition to variability in the program approaches, there was a large variability in evaluation approaches. Self-report surveys, pre-/post-tests, interviews, email questionnaires, and system approaches were all found to have been used. Self-report surveys and pre-/post-tests were used by shorter training programs and administered during or shortly after the completion of training. That period was not enough for such training to have had an impact on participants, but rather they reported on the perception of participants about the course and whether changes in knowledge have occurred. Longer trainings, on the other hand, were more likely to follow-up participants through implementation of research projects over which additional technical assistance and mentorship are provided. Specific deliverables for most of those training programs which include writing a protocol and/or writing and publishing a manuscript enable them to determine the level of their success. Understandably, the long period of implementation, in addition to both technical and financial support provided to complete research projects, is likely to increase the number of protocols written, research projects conducted and published, and the influence in policy and practice change among others. However, much needs to be done to fully understand the impact of such capacity strengthening trainings. For instance, better baseline assessment using comprehensive tools, such as those employed by systems approach [25], are needed as well as better reporting on whether there were other outside enabling factors.

The evaluation metrics for research capacity strengthening programs are debated in the literature. Some suggest that success should be measured in terms of papers published [26]; however, this implies that writing a paper is the ultimate goal for the training or target competency desired by the individual. Others advise that change in policy and practice should be the end goal of research capacity strengthening activities in order to improve the quality of service delivery [27]. There are few training programs that cover all necessary competencies to write and publish a research paper as an indicator of success; this requires not only substantial resources in terms of trainers and mentors, time, and money, but also strong candidates, thus limiting the number of training participants. Further, using research to change policy is difficult, requiring ongoing engagement and co-operation between all stakeholders, and documenting such change in a concrete and objective way is even more challenging. Alternatively, Harries et al. [21] advocates for embedding research training activities into existing health programs. This suggests that training is budgeted for as any other activity of the program and often times participants in that training are staff who work within health programs.

The challenges to research capacity strengthening identified in this review have been observed by others. Several studies report limited funding for research [6, 8, 26, 28, 29], no dedicated time for research [3, 26, 29], and a lack of mentorship and institutional support [8, 13, 27]. In addition, challenges identified but not discussed in papers in this review include difficulties in carrying out quality evaluation particularly for long term outcomes and the imbalanced focus on research methods and process at the expense of research advocacy, promotion, negotiation, and resource mobilization [30]. These challenges are complex and call for sustainable partnerships and commitment to the goals of research capacity strengthening in Africa.

While academic and non-academic training programs face similar challenges, some of the challenges, such as lack of institutional support or research leadership, are more pronounced in non-academic settings. Our review identified one program with institutional support [13] which also had the most significant and quantified impact on society through policy and practice changes. We believe that the research developed as part of academic trainings is more likely to be published because of the existence of such support. Furthermore, trainees in academic programs tend to have time separated out for research and thus do not face the similar challenge of balancing work and research training concurrently. Academic programs may also be appealing because of the existing accreditation process that is difficult for the non-academic program.

There were two primary limitations to this systematic review. First, for the articles identified, relevant information on important features, including features that would support replicability, were missing. For example, it is possible that some programs offered on-going mentorship, but we were unable to report this feature because it was not described in the paper. Information on financial and material resources, qualifications, and number of trainers/facilitators needed to undertake capacity strengthening activities were poorly reported, which not only is a limitation of this review, but may weaken the ability to replicate the program in other settings. A second limitation is that this review only included scientific articles that had the abstract and full paper available in English. Therefore, we believe that programs published in languages other than English or presenting their results in grey literature may have been overlooked. Though grey literature may offer more detailed information about training programs, their use is hampered by the difficulty in accessing reports years after their production and limited information on the individuals involved in producing the report. However, because of publication bias in scientific literature, this review may have missed training programs that were deemed less successful, less “innovative”, or may have had less academic collaboration. On the other hand, the limited number of articles and the limited detail in the articles serves as a call-to-action for individuals developing and leading such research capacity strengthening activities to ensure that approaches and lessons learnt are shared more widely and with enough details to facilitate the replication of their activities in other settings.

## Conclusion

Research capacity strengthening activities through non-academic trainings can generate researchers capable of developing research question through to publication and integration of findings into policy and practice. Institutional support, increased funds, and dedicated time for research activities are critical factors that lead to development of successful health research capacity strengthening programs. Achieving representation of African authors in scientific health literature may rely in part on the outcome of research capacity strengthening programs. However, few publications examine this in a robust way or with sufficient detail for replication. Replication of successful models relies on robust evaluation methods and program documentation made accessible in the peer-reviewed literature. We thus recommend further research into feasible methods of tracking medium term and long term research impact. Further, future reviews could explore research capacity strengthening trainings in other regions.
